# Phenotypically Similar Rare Disease Identification from an Integrative Knowledge Graph for Data Harmonization: Preliminary Study

**DOI:** 10.2196/18395

**Published:** 2020-10-02

**Authors:** Qian Zhu, Dac-Trung Nguyen, Gioconda Alyea, Karen Hanson, Eric Sid, Anne Pariser

**Affiliations:** 1 Division of Pre-Clinical Innovation National Center for Advancing Translational Sciences (NCATS) National Institutes of Health (NIH) Rockville, MD United States; 2 ICF International Inc Rockville, MD United States; 3 Office of Rare Diseases Research (ORDR) National Center for Advancing Translational Sciences (NCATS) National Institutes of Health (NIH) Bethesda, MD United States

**Keywords:** GARD, rare diseases, phenotypical similarity, data harmonization

## Abstract

**Background:**

Although many efforts have been made to develop comprehensive disease resources that capture rare disease information for the purpose of clinical decision making and education, there is no standardized protocol for defining and harmonizing rare diseases across multiple resources. This introduces data redundancy and inconsistency that may ultimately increase confusion and difficulty for the wide use of these resources. To overcome such encumbrances, we report our preliminary study to identify phenotypical similarity among genetic and rare diseases (GARD) that are presenting similar clinical manifestations, and support further data harmonization.

**Objective:**

To support rare disease data harmonization, we aim to systematically identify phenotypically similar GARD diseases from a disease-oriented integrative knowledge graph and determine their similarity types.

**Methods:**

We identified phenotypically similar GARD diseases programmatically with 2 methods: (1) We measured disease similarity by comparing disease mappings between GARD and other rare disease resources, incorporating manual assessment; 2) we derived clinical manifestations presenting among sibling diseases from disease classifications and prioritized the identified similar diseases based on their phenotypes and genotypes.

**Results:**

For disease similarity comparison, approximately 87% (341/392) identified, phenotypically similar disease pairs were validated; 80% (271/392) of these disease pairs were accurately identified as phenotypically similar based on similarity score. The evaluation result shows a high precision (94%) and a satisfactory quality (86% F measure). By deriving phenotypical similarity from Monarch Disease Ontology (MONDO) and Orphanet disease classification trees, we identified a total of 360 disease pairs with at least 1 shared clinical phenotype and gene, which were applied for prioritizing clinical relevance. A total of 662 phenotypically similar disease pairs were identified and will be applied for GARD data harmonization.

**Conclusions:**

We successfully identified phenotypically similar rare diseases among the GARD diseases via 2 approaches, disease mapping comparison and phenotypical similarity derivation from disease classification systems. The results will not only direct GARD data harmonization in expanding translational science research but will also accelerate data transparency and consistency across different disease resources and terminologies, helping to build a robust and up-to-date knowledge resource on rare diseases.

## Introduction

A rare disease in the United States is defined by the 1983 Orphan Drug Act as a condition that affects fewer than 200,000 people [1], whereas the analogous legislation introduced in the European Union in 2000 considers a disease to be rare when it affects fewer than 1 in 2,000 people [2]. In comparison to common diseases, health care providers are challenged by a lack of familiarity with diagnosing and treating rare diseases, which can lead to missed, delayed, or inaccurate diagnoses even when an approved, effective therapy is available [3]. Improved understanding and recognition of rare diseases are key for accurate and timely diagnosis, and this relies on broad dissemination of and access to knowledge about rare diseases [4]. A huge amount of effort has been made to develop rare disease resources for patients, families, and clinicians, such as the Genetic and Rare Diseases Information Center (GARD) [5], Orphanet [6], and Monarch Disease Ontology (MONDO) [7]; however, disparate data and incomplete data harmonization are still major barriers to improved coordination across specialists, leading to inefficiencies and delays in diagnosis, care, and treatment. This is exemplified by the difficulty faced in accurately answering the question, *how many total rare diseases are there?* A recent report by Haendel et al [8], after an examination of multiple rare resources, concluded that “there are total of 10,393 rare diseases in MONDO…the majority, 6370 rare diseases, are presented in three or more resources, whereas 4023 are unique to one source.” The fact that more than one-third of rare diseases are unique to 1 source highlights a reality that those resources continue to use their own disease definitions or harmonization rules to develop their rare disease vocabularies. Insufficient effort put toward data harmonization ultimately leads to redundancy in categorization efforts and a resulting inconsistency of rare disease representation globally.

The goal of data harmonization is to improve the compatibility of data collected from independent sources (horizontally) in order to better understand disease etiology from different angles, which may forward the discovery of therapeutic approaches for rare diseases. For each individual source, data harmonization is crucial to better represent and organize data for supporting data harmonization horizontally. Current data harmonization efforts are primarily aligning standard nomenclatures or human efforts to translate specific medical and clinical features into a standardized and sharable format. For instance, Pontikos et al [9] introduced Phenooplis, an open platform for the harmonization and analysis of genetic and phenotypic data that harmonize phenotypes with the help of Human Phenotype Ontology (HPO). The International Cancer Genome Consortium (ICGC) and The Cancer Genome Atlas (TCGA) invited the cancer-genomics and bioinformatics communities to work together to identify the best pipelines for the detection of mutations in DNA-sequencing reads for cancer genomes in order to facilitate the harmonization of mutation-calling procedures among institutions [10,11]. Orphanet and OMIM (Online Mendelian Inheritance of Man) heavily relied on human efforts for their data curation and harmonization [12,13]. To avoid cumbersome human efforts and a lack of rare disease standards in this study, we proposed to systematically identify phenotypically similar rare diseases from GARD and determine their similarity types, including duplicate diseases, sibling diseases, and subtypes for supporting rare disease data harmonization.

Rare disease designations are often in conflict across different datasets due to the differing statutory requirements used in defining a rare disease in different countries, and as such, useful methods to improve interoperability across these broad terminologies and standards are required. With the aim of eliminating data redundancy and inconsistency across different resources, improving data interoperability, and facilitating data harmonization, the implementation of a knowledge graph is capable of semantically organizing and integrating complex networks of data into one collection. Knowledge graphs have been widely applied in the medical domain and in the rare disease field. For instance, Reumann et al [14] reported their solution for cognitive differential diagnosis (DDx) in rare diseases based on knowledge graph technology that incorporates data from ICD-10, DOID, medDRA, PubMed, Wikipedia, Orphanet, the CDC, and anonymized patient data. Li et al [15] presented their work to develop a rare disease classification algorithm established on a knowledge graph. Sosa et al [16] applied a knowledge graph–embedding method that explicitly models the uncertainty associated with literature-derived relationships and uses link prediction to generate drug repurposing hypotheses for rare diseases. In this study, we accessed data from an integrative knowledge graph that we developed from our previous study [17] with a variety of rare disease-related resources for phenotypical similarity identification among GARD diseases.

In this study, we report our preliminary work to identify phenotypically similar GARD diseases from an integrative knowledge graph using 2 approaches: (1) disease mapping comparison, and (2) phenotypical similarity derivation from disease classification systems. This effort will not only direct GARD data harmonization but will also support data harmonization across different resources, and eventually support clinical decision making. Phenotypically similar GARD diseases applied in this study specifically refer to disease subtypes and sibling diseases that share similar clinical manifestations. For example, 2 GARD diseases of “lactate dehydrogenase deficiency” and “lactate dehydrogenase A deficiency” are subtypes, and they have similar phenotypical profiles.

### Background and Materials

#### Rare Disease Resources

The Genetic and Rare Diseases Information Center (GARD) is a program managed by the National Center for Advancing Translational Sciences (NCATS), National Institutes of Health (NIH). Since 2003, GARD has provided the public with access to current, reliable, and easy-to-understand information about rare and genetic diseases [5]. As part of the data harmonization effort toward furthering the development of the GARD, we harmonized GARD diseases according to their phenotypical similarity in this study. To fulfill this task, we assessed phenotypical similarity among GARD diseases by leveraging several well-known disease resources, including Orphanet, OMIM, MONDO, the HPO, and the UMLS (Unified Medical Language System), owing to their complementary focus and coverage. We briefly describe these applied resources below.

Orphanet is an EU resource that focuses on gathering and improving knowledge on rare diseases [6]. Rare diseases in the Orphanet, depending on their clinical presentation, are included in as many classifications as needed. The Orphanet classification is organized according to three hierarchical levels: group of disorders, disorder, and subtype of a disorder. The disorder level is designated as the main topologic level for each clinical entity characterized by a set of homogeneous phenotypic abnormalities and evolution, allowing for a definitive clinical diagnosis [18,19].

OMIM (Online Mendelian Inheritance in Man**)** is a comprehensive, authoritative compendium of human genes and genetic phenotypes that is freely available and updated daily. It contains information on all known mendelian disorders and over 15,000 genes. OMIM focuses on the relationship between phenotype and genotype [20].

MONDO (Monarch Disease Ontology) aims to harmonize disease definitions across the world. It is a semi-automatically constructed ontology that merges multiple disease resources to yield a coherent merged ontology. One feature of the MONDO is that it curates precise 1-to-1 equivalence axioms connecting to other resources, validated by OWL reasoning [7]. MONDO provides a hierarchical structure that can be used for classification or for rolling up diseases to higher-level groupings.

The Human Phenotype Ontology (HPO) provides a standardized vocabulary of phenotypic abnormalities encountered in human disease. HPO currently contains over 13,000 terms and over 156,000 annotations to hereditary diseases [21].

The Unified Medical Language System (UMLS) is a terminology integration system developed at the National Library of Medicine (NLM). The UMLS Metathesaurus integrates more than 160 biomedical vocabularies. Synonymous terms from the various source vocabularies are grouped into one concept [22].

#### An Integrative Knowledge Graph

We previously developed an integrative knowledge graph with 34 different biomedical data resources at the time of writing, including the aforementioned resources. This graph database is hosted in Neo4j and is publicly accessible without login credentials [17]. In this study, we accessed this knowledge graph to obtain data from the aforementioned resources and applied it for the measurement of phenotypical similarity among GARD diseases.

## Methods

**Figure 1:**
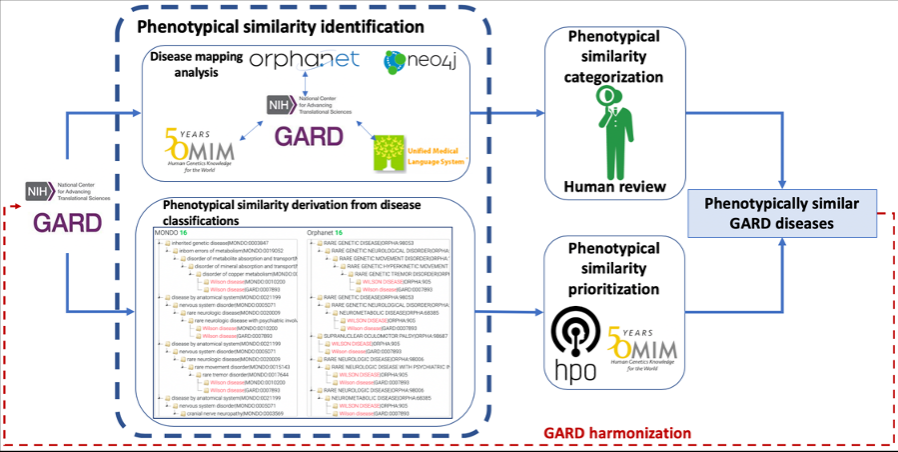
The architecture of phenotypically similar GARD disease identification.

In this study, we aimed to identify phenotypical similarity among rare diseases to support data harmonization and data interoperability with existing standardized terminologies and ontologies. We designed two complementary approaches: (1) analysis of disease mappings to Orphanet, OMIM, and the UMLS to measure phenotypical similarity among GARD diseases; (2) prioritizing phenotypical similarity derived from MONDO and Orphanet disease classification systems with shared phenotypes from the HPO and genes from OMIM. The architecture of this study is shown in Figure 1.

### Phenotypical Similarity Identification Based on GARD Disease Mappings

In order to identify phenotypical similarity, we computed disease similarity among disease mappings between GARD diseases and disease concepts from Orphanet, OMIM, and the UMLS, which offer a wide spectrum of characteristics of rare diseases—in Orphanet, diseases are defined upon their clinical presentation; in OMIM, disease definition is based on genetic etiology; in UMLS, a broader biomedical definition of diseases is offered.

### Disease Mapping Retrieval from the Knowledge Graph

We obtained disease mappings from the aforementioned knowledge graph. There are 2 ways to retrieve disease mappings for GARD diseases from the knowledge graph: (1) by developing mappings based on specific edge properties; for instance, 2 concepts with the same concept names are mapped via one edge property of “N_Name”; (2) by extracting mappings directly from GARD disease nodes, which store GARD-curated external mappings to Orphanet, OMIM, and the UMLS. To ensure mapping quality, we performed the second approach by accessing 1 node property of I_CODE and storing external mappings for each GARD disease node. For instance, 3 external mappings, including “OMIM:603358,” “ORPHANET:53693,” and “UMLS:C1864002” for the GARD disease of “GRACILE SYNDROME(GARD:0000001),” are stored in its property of “I_CODE” and can be retrieved by executing the following Cypher Query 1 [23], which is Neo4j's graph query language that allows users to store and retrieve data from the graph database:

**Cypher Query 1.** match P = (n:S_GARD^a^) where any (x in n.I_CODE where x= “GARD:0000001”) return n.I_CODE

^a^S_GARD referring to GARD data

We executed the Cypher Queries listed in Table 1 to retrieve disease mappings for GARD diseases. Each GARD disease obtains zero to multiple mappings accordingly. For instance, “Gracile Syndrome (GARD:0000001)” has the 3 disease mappings described above; however, “Acalvaria ( GARD:0000361)” only has 1 mapping, “ORPHANET:945.” To ensure that each GARD disease was associated with at least 1 mapping for similarity measurement, we excluded 1498 GARD diseases with no mappings to any of these 3 resources.

**Table 1:** Disease mapping extraction from the Neo4j knowledge graph.

Disease mappings	Cypher Queries
GARD2Orphanet	match P = (n:S_GARD) where any (x in n.I_CODE where x=~ ‘ORPHA.*’)return distinct n.I_CODE
GARD2OMIM	match P = (n:S_GARD) where any (x in n.I_CODE where x=~ ‘OMIM.*’)return distinct n.I_CODE
GARD2UMLS	match P = (n:S_GARD) where any (x in n.I_CODE where x=~ UMLS.*’)return distinct n.I_CODE

### Calculating Similarity to Prioritize Phenotypical Similarity of GARD Disease Pairs

In order to compare phenotypical similarity among the GARD diseases based on their similarity, we enumerated all mappings obtained for 5236 GARD diseases and ended with a total of 9672 mappings. For each GARD disease, we generated fingerprints based on those mappings. One disease mapping corresponding to one binary fingerprint, with presence denoted as 1 and absence denoted as 0. To this end, each GARD disease was represented as a vector of 9672 bits. Then, we calculated cosine similarity [24] for each pair of GARD diseases based on their fingerprints. For those disease pairs without any shared mappings, which means their similarity score equals 0, we excluded them for manual similarity identification.

### Phenotypically Similar GARD Disease Identification

To determine the phenotypical similarity of GARD diseases, our subject matter experts (GA, KH, and ES) manually evaluated the prioritized disease pairs based on their similarity scores generated from the above step. The manual validation was not only attempting to examine the accuracy of computational results to establish business rules for further GARD data harmonization, but also to validate correctness and coverage of the GARD-curated external mappings.

The manual review process consisted of 3 steps: (1) categorizing GARD disease pairs to phenotypical similarity types, namely “Duplicates,” “Subtypes,” “Siblings,” and “Unrelated;” (2) researching the latest epidemiology studies (eg, PubMed articles, trusted resources) for each disease if applicable, to re-evaluate the qualification of RARE disease based on the US definition of rare disease [1]; (3) documenting the decision-making process for future reference. As an example demonstrating this review process, “Testicular Cancer (GARD:0007746)” and “Testicular germ cell tumor (GARD:0013047),” with a similarity score of 0.71, were initially grouped as subtypes. However, researching the latest epidemiological data for testicular cancer uncovered that “in 2017, there were an estimated 269,769 men living with testicular cancer in the United States” [25]; this indicates that the prevalence rate of testicular cancer does not meet (ie, is higher than) the US definition of rare diseases, and so it was marked to “Retire.”

In this context, we defined *precision* as the fraction between the number of correctly identified phenotypically similar disease pairs based on manual evaluation and the total number of similar disease pairs identified; we defined *recall* as the fraction between the number of correctly identified phenotypically similar disease pairs and the total number of similar disease pairs; we defined *F measure* as the balanced harmonic mean of the precision and recall. We computed precision, recall, and F measure to measure the performance of this approach.

### Phenotypical Similarity Derivation from Disease Classification Systems

**Figure 2:**
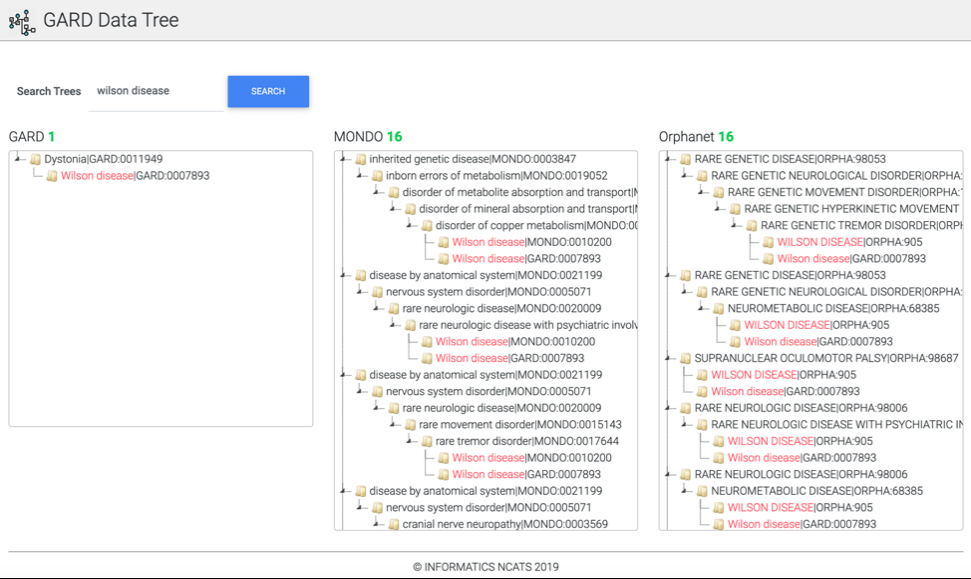
Disease tree visualization via the GARD Data Tree web tool.

Diseases from the same disease category exhibit a high phenotypic homogeneity [26]; we assume that phenotypical similarity is evidently presenting among sibling diseases, which share the same parent diseases in disease classification systems. To further prove our assumption by assessing 3 disease classification systems, including GARD, MONDO, and Orphanet, we developed a web application to search and review a specific disease term presenting in these 3 disease trees to perform a comparison. This web application is publicly accessible [27]. Figure 2 shows one screenshot of the search results for “Wilson disease.” MONDO and Orphanet have more refined and complete disease classifications than the GARD, which enables phenotypical similarity identification for GARD diseases.

### Retrieving Phenotypically Similar GARD Diseases

With the help of the GARD Data Tree web tool, we were able to form a process of deriving phenotypical similarity among GARD diseases in 3 steps: (1) mapping GARD diseases to MONDO and Orphanet; (2) extracting all sibling diseases of the mapped MONDO and Orphanet diseases from their disease trees; and (3) mapping the retrieved sibling diseases back to the GARD. The GARD diseases retrieved from the third step should be phenotypically similar to the query GARD disease from the first step. We further validated them by leveraging their associated phenotypes and genotypes.

**Figure 3:**
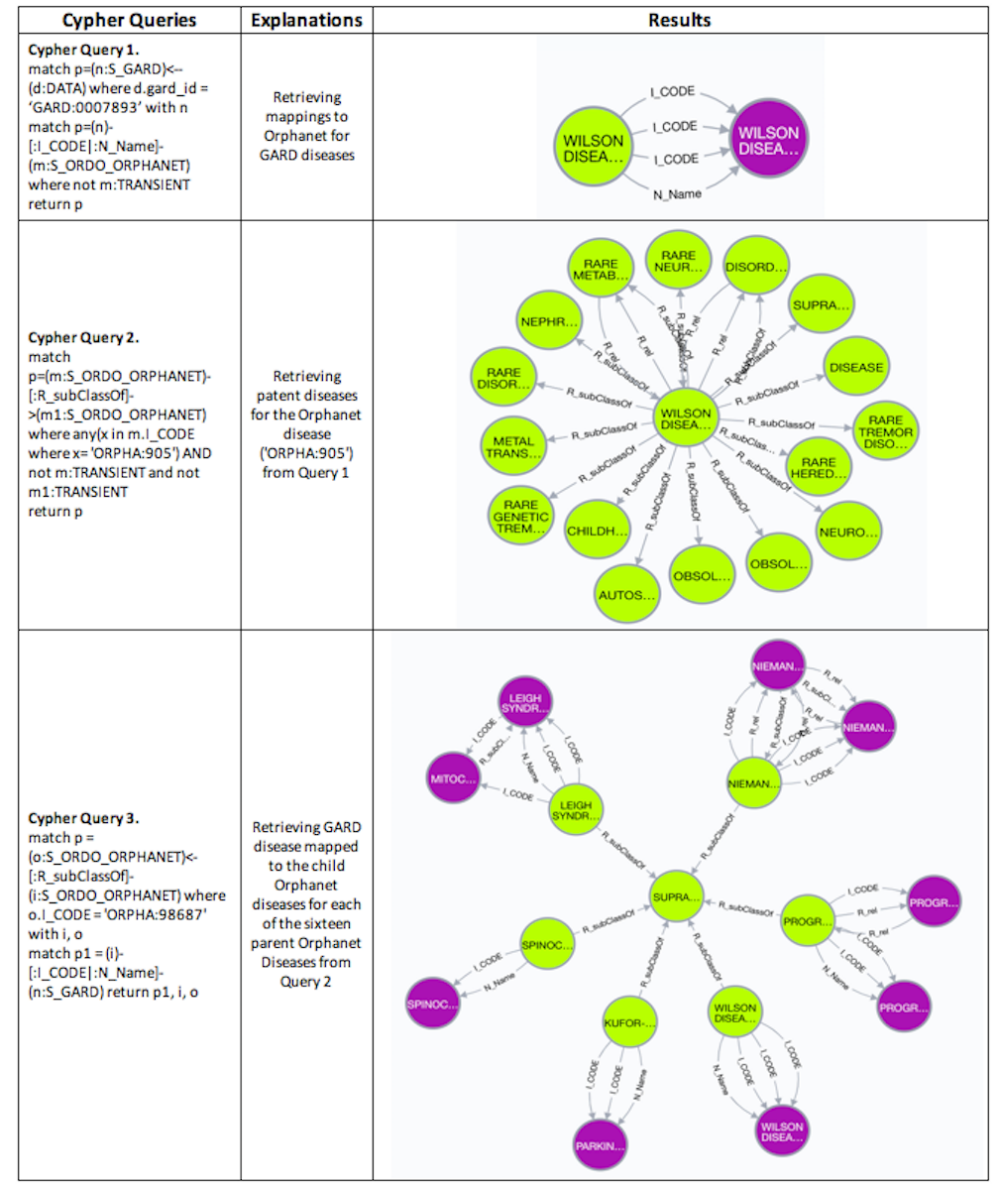
Cypher query examples for extracting phenotypically similar GARD diseases by navigating Orphanet disease classification systems.

These 3 steps can be formalized in Cypher Queries accordingly; examples are shown in Figure 3. After obtaining mappings between GARD and Orphanet/MONDO by executing Cypher Query 1 shown in Figure 3, we searched parent diseases of those mapped MONDO and Orphanet diseases. Cypher Query 2 is an example of extracting Orphanet parent diseases for the Orphanet concept “Wilson Disease (ORPHA:905),” which is mapped to “GARD:0007893” from Cypher Query 1. Cypher Query 3 demonstrates a process that extracts all child diseases for 1 Orphanet parent disease, “SUPRANUCLEAR EYE MOVEMENT DISORDER (ORPHANET:98687),” which is 1 parent node returned from Cypher Query 2, and maps those child Orphanet diseases to GARD diseases. In order to identify the most phenotypically similar GARD diseases obtained from Cypher Query 3 to the inquiry disease “Wilson Disease (GARD:0007893),” we prioritized similarity based on their associated phenotypes and genes.

### Prioritizing Phenotypically Similar GARD Diseases Based on Phenotypes and Genotypes

Given the fact that a majority of rare diseases are genetic in origin and that clinical phenotypes are one of the red flags increasing rare disease attentiveness in clinical practice [28], we developed a protocol for prioritizing phenotypical similarity based on phenotypes and genotypes. We collected phenotypes from the HPO and genes from OMIM from our knowledge graph, for those similar GARD disease pairs identified from the above step. The number of phenotypes and genes shared by each pair of phenotypically similar GARD diseases was applied for prioritization.

## Results

### Results of Disease Mapping Analysis

#### Disease Concept Retrieval

We extracted disease mappings between GARD and Orphanet, OMIM, and the UMLS from our Neo4j knowledge graph. The retrieval results are shown in Table 2.

**Table 2:** Results of disease mapping retrieval from Neo4j graph.

Types of mapping	Number of mappings
GARD2Orphanet	2,869
GARD2OMIM	3,500
GARD2UMLS	3,584

#### Disease Similarity Calculation

We enumerated disease pairs for 5236 GARD diseases with disease mappings and calculated cosine similarity for those GARD pairs. After excluding those disease pairs with similarity equaling 0, 392 diseases pairs remained. Table 3 summarizes the results of the similarity calculation.

**Table 3:** Similarity calculation results for disease pairs (n=392).

Similarity scores	Number of disease pairs
1	34
0.5 <= Similarity < 1	264
0 < Similarity < 0.5	94

#### Evaluation and Disease Similarity Identification

Our subject matter experts manually reviewed these 392 disease pairs and assigned their similarity types accordingly. Table 4 shows their review results.

Of the 392 disease pairs, 341 (87%) were identified and categorized as phenotypically similar, corresponding to the categories “Duplicated,” “Siblings,” and “Subtypes.” Of those 341 disease pairs, 271 disease pairs (80%) with similarity scores greater than 0.5 were verified as phenotypically similar. However, 34 disease pairs were determined to be “Unrelated,” and another 17 disease pairs were “Ungrouped;” this needs further discussion, and so we excluded the latter group for calculations of precision, recall, and F measure.

**Table 4:** Manual review results for the disease pairs (n=392); precision=94%, recall=79%, F measure=86%.

Variables			Phenotypical similarity types							
			Duplicated	Siblings		Subtypes		Unrelated		Ungrouped
**Number of disease pairs, n**										
	Phenotypically similar (n=341)		105	117	119		N/A^a^		N/A	
	Not phenotypically similar (n=51)		N/A	N/A	N/A		34		17	
**Similarity scores, n**										
	**Phenotypically similar (n=341)**									
		0.7≥Score≥1 (n=95)	47	21		27		N/A		N/A
		0.5≥Score≥0.7 (n=176)	42	81		53		N/A		N/A
		Score>0.5 (n=70)	16	15		39		N/A		N/A
	**Not phenotypically similar (n=51)**									
		0.7≥Score≥1 (n=16)	N/A	N/A		N/A		8		8
		0.5≥Score≥0.7 (n=15)	N/A	N/A		N/A		8		7
		Score>0.5 (n=20)	N/A	N/A		N/A		18		2

^a^N/A: not applicable.

### Results of Phenotypical Similarity Derivation from Disease Classification Systems

**Figure 4:**
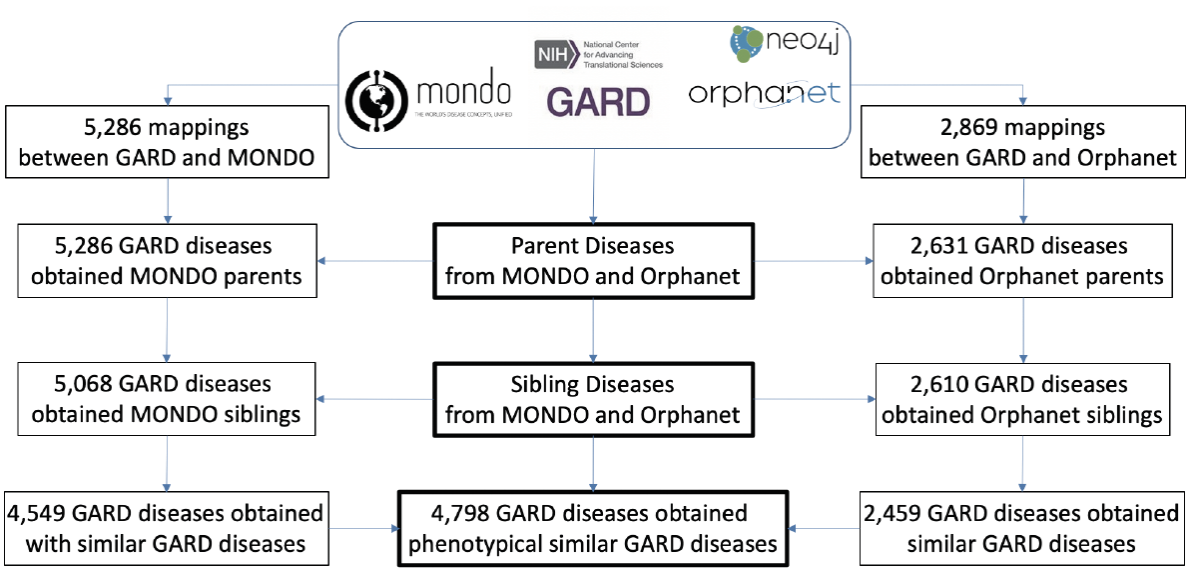
Results of phenotypically similar GARD disease retrieval based on MONDO and Orphanet disease classifications.

Based on the above analysis, 53 GARD diseases were marked for retirement. Of the remaining of 5955 GARD diseases, 4798 GARD diseases obtained 1 or more phenotypically similar GARD disease(s) from this step. The stepwise results are shown in Figure 4.

Of 5286 GARD diseases mapped to one of 21,823 MONDO diseases with parent diseases, 4549 GARD diseases obtained phenotypically similar GARD diseases via MONDO sibling disease mappings. Of 2631 GARD diseases mapped to one of 7024 Orphanet diseases with parent diseases, 2459 GARD diseases obtained phenotypically similar GARD diseases via Orphanet sibling disease mappings. By combining these 2 lists of mappings, 4798 GARD diseases obtained phenotypically similar diseases. We paired these 4798 GARD diseases with identified phenotypically similar diseases and ended with unique 241,604 GARD disease pairs.

#### Phenotypically Similar Disease Prioritization Based on Phenotypes and Genotypes

Of the 241,604 disease pairs identified for these 4798 GARD diseases, 84,054 disease pairs shared at least 1 phenotype and 396 disease pairs shared at least 1 gene. By combing these 2 sets, there are 360 GARD disease pairs with at least 1 shared phenotype and gene. As all of those disease pairs were extracted from sibling diseases presenting in the MONDO and Orphanet, these 360 disease pairs were consequently grouped as “Siblings” with different degrees of phenotypical similarity based on the number of their shared phenotypes and genes.

By combining 341 disease pairs identified from the step of disease mapping analysis, 662 disease pairs showed phenotypical similarity. It is worth noting that there are 39 overlaps between these 2 sets. Based on the manual evaluation shown in Table 4, these 39 pairs consist of 25 disease pairs that are sibling diseases, 7 disease pairs that are subtypes, 2 pairs that are duplicates, and 5 pairs that are unrelated diseases.

## Discussion

In this study, we identified and prioritized phenotypical similarity among GARD diseases by comparing disease similarity and deriving phenotypical similarity from disease classification systems. As a proof-of-concept, we demonstrated the usefulness of the identified phenotypically similar disease pairs to support data harmonization for GARD. By incorporating these identified similar diseases, GARD will have the capability of supporting education and clinical decision making; for instance, GARD can provide more complementary information not only for the inquiry disease but also for phenotypically similar diseases.

There are many different rare disease resources available, and each of them has their own strength and focus. OMIM classifies diseases based on their genetic cause, Orphanet defines rare diseases based on phenotypical characteristics, and UMLS incorporates biomedical vocabulary and standards to define their disease concepts. Given the complementary definition of disease concepts from these 3 resources, we employed their mappings to the GARD diseases for disease similarity comparison. Of the 392 disease pairs, 271 disease pairs (80%) with similarity scores greater than 0.5 were successfully validated as clinically relevant by our genetic specialists. Besides these true positives, feedback from our subject matter experts on the false positives [ie, 16 disease pairs (~4%) with similarity scores greater than 0.5 were manually determined as irrelevant] and false negatives [ie, 70 disease pairs (~18%) with similarity scores less than 0.5 were manually determined as relevant] illustrates that it is important to accurately capture the latest information in regard to disease mappings across different resources, and to incorporate human interpretations. For example, “Spondylothoracic dysostosis (GARD:0006798)” and “Spondylocostal dysostosis 1 (GARD:0010726)” share 3 of the same mappings, “ORPHA:2311,” “UMLS:C0265343,” and “OMIM:277300,” so their similarity score equals 1.0, indicating that they should be highly similar. However, our experts marked them as “Unrelated” due to the fact that these 2 conditions were grouped together in the past (both were previously referred to as Jarcho-Levin syndrome); they are considered as distinct conditions now, according to references from GHR (Genetic Home Reference) [29,30]. Berdon et al [31] also discussed the clinical and radiological distinction between these 2 diseases. Another example is “Hunter Carpenter Macdonald syndrome (GARD:0002751)” and “Infantile neuroaxonal dystrophy (GARD:0003957),” which have a similarity score of 0.35, indicated they should be less relevant. However, it was marked as relevant by our experts given that PLA2G6-associated neurodegeneration (PLAN) comprises a continuum of 3 phenotypes with overlapping clinical and radiologic features for these 2 diseases, and similar evidence can be found at Orphanet [32] that reveals that Hunter-Carpenter-McDonald syndrome has been moved to “Infantile neuroaxonal dystrophy.” In comparison of the total 13,705,230 GARD disease pairs, there are only 392 disease pairs with similarity scores greater than 0, which might direct the extension in 2 ways. First, 3 selected resources might not be comprehensive enough to cover all GARD diseases for disease similarity comparison based on their disease mappings. Therefore, we plan to extend our work with additional rare disease resources, such as MONDO, Disease Ontology, NCI Thesaurus, etc. Second, external disease mappings curated by GARD are accurate but might be incomplete due to cumbersome human effort. Thus, we will extend the disease mappings by inferring new associations via network analysis from the Neo4j knowledge graph.

Phenotypical similarity derivation from disease classifications resulted in 360 disease pairs shared with at least 1 phenotype and gene, and they are grouped as sibling diseases. Among 241,604 disease pairs retrieved from the disease classification trees, there are 84,054 disease pairs that share at least 1 phenotype and 396 disease pairs that share at least 1 gene. Compared to the number of disease pairs with shared phenotypes, a relatively small number of disease pairs shared at least 1 gene; we are planning to obtain more genes for GARD diseases from other resources, including DisGeNet [33] and ClinVar [34]. Given the success we gained from this study in identifying phenotypical similarity derived from sibling diseases from disease classifications, we propose to extend this work with subtype diseases (ie, parent diseases and child diseases) by mining disease classifications. Once we have GARD diseases that we are able to assign to those relevant categories, we will develop our own disease classification system, which will not only define more accurate disease definitions and relationships among those diseases but will also serve as a unique, rare disease resource in the United States.

By combining 2 sets generated by our 2 approaches, we identified 662 phenotypically similar disease pairs and mapped them to 4 phenotypical similarity types, namely, “Duplicates,” “Subtypes,” “Siblings,” and “Unrelated,” which will be applied to direct GARD data harmonization. To be specific, for “Duplicate” disease pairs, we will select and keep primary diseases in the GARD database; “Siblings” and “Subtypes” will direct GARD disease classification regeneration; for “Unrelated” diseases, we will keep these 2 diseases separately in the GARD database.

By comparing these 2 sets, there are 39 overlapped disease pairs. These 39 disease pairs were grouped as “Siblings” by the second approach of disease classification derivation. However, based on the evaluation result (Table 4) from the first approach of disease mapping analysis, of these 39 disease pairs, 25 disease pairs were grouped as “Siblings,” 7 pairs were grouped as “Subtypes,” 2 pairs were grouped as “Duplicated,” and 5 pairs were grouped as “Unrelated.” For instance, “Malignant hyperthermia” and “King Denborough syndrome” are classified as sibling diseases by the second approach, since they are siblings in Orphanet, which groups them under the same disease parent class of “Rare Disease With Malignant Hyperthermia (ORPHA:466658).” However, they are determined as different diseases by our subject-matter experts, and the same statement has been made in the GARD page for “King-Denborough syndrome (GARD:0008433),” claiming that “King-Denborough syndrome is a congenital myopathy associated with susceptibility to malignant hyperthermia (GARD:0006964)” [35]. Such discrepancies occurring across different resources unveiled from this study illustrate that there is an urgent need to propose a standard protocol for guiding data harmonization in the rare disease field globally. Regardless of phenotypical similarity types, the process our subject-matter experts took in the evaluation step is crucial to re-evaluate rare diseases with the latest prevalence data, which is one critical step to determine their eligibility of RARE. For instance, there are more than 200,000 individuals in the United States who are affected with familial Alzheimer disease (GARD:0000632) [36,37]; thus, the prevalence rate of this disease does not meet the criteria of the United States’ rare disease definition, so it will be retired from the GARD database.

### Conclusion

In this paper, we report our recent effort at identifying phenotypical similarity among rare diseases by leveraging disease mappings among various resources and disease classifications. This effort will not only direct further GARD data harmonization but will also highlight the value of cross-resource collaboration. We propose to extend this work with more rare disease resources at the NIH or outside the NIH for the improved assembly of information for rare diseases in order to better disseminate information to patients and health care providers.

## Abbreviations

GARD: genetic and rare diseases
HPO: Human Phenotype Ontology
MONDO: Monarch Disease Ontology
OMIM: Online Mendelian Inheritance in Man
UMLS: Unified Medical Language System
